# Conformational alterations in the CD4 binding cavity of HIV-1 gp120 influencing gp120-CD4 interactions and fusogenicity of HIV-1 envelopes derived from brain and other tissues

**DOI:** 10.1186/1742-4690-8-42

**Published:** 2011-06-02

**Authors:** Lachlan Gray, Jasminka Sterjovski, Paul A Ramsland, Melissa J Churchill, Paul R Gorry

**Affiliations:** 1 Center for Virology, Burnet Institute, Commercial Rd, Melbourne 3004, Australia; 2 Department of Biochemistry and Molecular Biology, Monash University, Wellington Rd, Clayton 3800, Australia; 3 Center for Immunology, Burnet Institute, Commercial Rd, Melbourne 3004, Australia; 4 Department of Immunology, Monash University, Commercial Rd, Melbourne 3004, Australia; 5 Department of Surgery (Austin Health), University of Melbourne, Studley Rd, Heidelberg 3084, Australia; 6 Department of Microbiology, Monash University, Wellington Rd, Clayton 3800, Australia; 7 Department of Medicine, Monash University, Commercial Rd, Melbourne 3004, Australia; 8 Department of Microbiology and Immunology, University of Melbourne, Royal Pde, Parkville 3010, Australia

## Abstract

**Background:**

CD4-binding site (CD4bs) alterations in gp120 contribute to HIV-1 envelope (Env) mediated fusogenicity and the ability of gp120 to utilize low levels of cell-surface CD4. In a recent study, we constructed three-dimensional models of gp120 to illustrate CD4bs conformations associated with enhanced fusogenicity and enhanced CD4-usage of a modestly-sized panel of blood-derived HIV-1 Envs (n = 16). These conformations were characterized by a wider aperture of the CD4bs cavity, as constrained by the inner-most atoms at the gp120 V1V2 stem and the V5 loop. Here, we sought to provide further validation of the utility of these models for understanding mechanisms that influence Env function, by characterizing the structure-function relationships of a larger panel of Envs derived from brain and other tissues (n = 81).

**Findings:**

Three-dimensional models of gp120 were generated by our recently validated homology modelling protocol. Analysis of predicted CD4bs structures showed correlations between the aperture width of the CD4bs cavity and ability of the Envs to mediate cell-cell fusion, scavenge low-levels of cell-surface CD4, bind directly to soluble CD4, and bind to the Env mAb IgG1b12 whose epitope overlaps the gp120 CD4bs. These structural alterations in the CD4bs cavity were associated with repositioning of the V5 loop.

**Conclusions:**

Using a large, independent panel of Envs, we can confirm the utility of three-dimensional gp120 structural models for illustrating CD4bs alterations that can affect Env function. Furthermore, we now provide new evidence that these CD4bs alterations augment the ability of gp120 to interact with CD4 by increasing the exposure of the CD4bs.

## Findings

The human immunodeficiency virus type 1 (HIV-1) envelope glycoproteins (Env) mediate virus entry into cells and exist as trimers, comprising the surface gp120 glycoproteins noncovalently linked to transmembrane gp41 glycoproteins that embed the complex into the viral membrane [1-3]. HIV-1 entry is initiated by gp120 binding to cellular CD4, which facilitates the initial attachment of virus to the target cell [4]. The binding of gp120 to CD4 results in dramatic conformational changes in gp120 that expose the binding site for a secondary coreceptor, which is either of the chemokine receptors CCR5 or CXCR4 (reviewed in [5-7]).

Crystallographic and biochemical studies of gp120 have provided valuable insights into mechanisms involved in CD4 binding and CD4-induced conformational changes [3,8-12]. The unliganded gp120 core of simian immunodeficiency virus (SIV) consists of a highly conserved inner domain which faces the trimer axis and a heavily glycosylated, globular outer domain which is mostly exposed on the surface of the trimer [8]. However, thermodynamic and structural analysis of the gp120-CD4 interaction demonstrated little evidence of a structured CD4 binding pocket on the unliganded gp120, and that CD4bs elements which influence gp120-CD4 affinity are formed from conformational alterations that occur after gp120 has encountered CD4 [2,10]. CD4 interacts with gp120 via surface-exposed residues within three separate regions distributed over six segments of gp120. These regions include the α-helices of the inner domain, the CD4 binding loop of outer domain, and the β20-β21 ribbon which becomes part of the gp120 bridging sheet, which is a structural element of gp120 formed after CD4 binding that is involved in coreceptor binding [3,11].

Changes in CD4 binding to gp120 contribute to different pathophysiological phenotypes of HIV-1, including the fusogenic properties of the Env [13,14]. Env mediates most of the acute cytopathic effects of HIV-1 infection in cultured cells [15], and membrane fusion appears to be an important factor contributing to HIV-1 cytopathicity *in vitro *[16,17]. In addition, enhancement of pathogenicity of chimeric simian-HIV (SHIV) strains in macaques frequently results from increased Env-mediated fusogenicity [18-22]. Moreover, the cytopathic effects of Env-mediated HIV-1 fusogenicity are evident in humans. For example, the presence of multinucleated giant cells in brain, formed by Env-mediated fusion between infected and uninfected macrophage lineage cells, is characteristic of HIV-1 encephalitis and a neuropathological hallmark of HIV-associated dementia [23].

To better understand the molecular mechanisms contributing to alterations in CD4 binding by primary gp120 proteins and the subsequent influence on Env function, we recently developed and validated a protocol to produce and utilize three-dimensional structural models of gp120 to deduce CD4bs alterations that influence CD4 binding and Env-mediated fusogenicity [13]. Using a modestly-sized panel of blood derived Envs (n = 16), we showed that a wider aperture of the predicted CD4bs cavity, as constrained by the inner-most atoms at the gp120 V1V2 stem and the V5 loop, contributed to increased fusogenicity and ability of gp120 to bind CD4. In the present study, we sought to provide further validation of the utility of these molecular models for understanding mechanisms that influence Env function, by characterizing, for the first time, the structure-function relationships of a larger panel of Envs derived from brain and other tissues (n = 81).

### Production and characterization of a panel of primary Env clones

**Figure 1:**
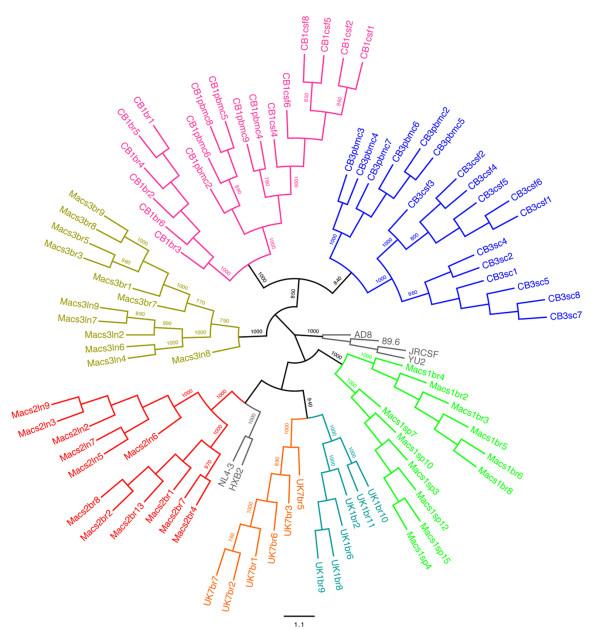
**Phylogenetic analysis of *env *nucleotide sequences**. The phylogenetic tree was constructed from an *env *nucleotide multiple sequence alignment using a maximum likelihood algorithm, as described previously [39]. The nucleotide sequences of HIV-1 AD8, 89.6, JRCSF, YU2, NL4-3 and HXB2 *env *genes were included for comparison. Numbers associated with each branch are bootstrap values obtained from 1000 replicates. Only values above 700 for the major branches are shown. Branch lengths are proportional to the amount of sequence divergence.

Primary HIV-1 viruses isolated from autopsy brain and/or cerebrospinal fluid, spinal cord, lymph node, spleen or PBMC from subjects CB1, CB3, MACS1, MACS2, MACS3, UK1 and UK7 have been described in detail previously [14,24-27]. The clinical characteristics of the subjects and coreceptor usage profiles of the primary viruses are summarized in Table 1. A 2.1 kb fragment spanning the KpnI to BamHI restriction sites in HIV-1 *env *(corresponding to nucleotides 6348 to 8478 in HXB2) was amplified from viral cDNA by PCR and cloned into the pSVIII-HXB2 Env expression vector [28], as described previously [29-33]. Between 4 and 6 functional Envs from each virus were identified by entry assays in JC53 cells with Env-pseudotyped GFP reporter viruses, as described previously [30,33-35] (Table 1). The coreceptor specificity of the cloned Envs was determined by entry assays in Cf2th-CD4/CCR5 and Cf2th-CD4/CXCR4 cells [35,36] with Env-pseudotyped luciferase reporter viruses, as described previously [29,35], which recapitulated the coreceptor usage of the primary viruses (Table 1). The Envs were sequenced in their entirety and subjected to multiple sequence alignments (data not shown) and phylogenetic analysis (Figure 1), which together showed that the Envs were independent and compartmentalized according to their tissue of origin. Thus, we established and characterized a new panel of Envs (n = 81) derived from autopsy brain and other tissues of 7 subjects who died from AIDS.

**Table 1:** Study subjects, HIV-1 isolates, and summary of Env phenotypes

Subject	Risk factor	Last CD4 count (cells/μl)	Antiretroviral(s)	HIV-1 encephalitis	Tissues yielding HIV-1 isolates	Name of virus isolate	Coreceptor usage of virus isolate	Envs cloned from virus isolate (n)	Functional	Coreceptor usage of cloned Envs
CB1	MH	10	ddI (prior AZT)	Severe	Brain	CB1-BR	X4	6	Yes	All X4
					CSF	CB1-CSF	R5	6	Yes	All R5
					PBMC	CB1-PBMC	R5	6	Yes	All R5
CB3	MH	5	ddI (prior AZT and ddC)	Severe	S.Cord	CB3-SC	R5	6	Yes	All R5
					CSF	CB3-CSF	R5	6	Yes	All R5
					PBMC	CB3-PBMC	R5	6	Yes	All R5
MACS1	MH	2	None	Severe	Brain	Macs1-BR	R5X4	6	Yes	All R5X4
					Spleen	Macs1-Spln	R5X4	6	Yes	All R5X4
MACS2	MH	52	AZT	Moderate	Brain	Macs2-BR	R5	5	Yes	All R5
					L.Node	Macs2-LN	R5	6	Yes	All R5
MACS3	MH	95	None	Moderate	Brain	Macs3-BR	R5	6	Yes	All R5
					L.Node	Macs3-LN	R5	6	Yes	All R5
UK1	IVDU	87	ddC (1 mo)	Moderate	Brain	UK1-BR	R5	4	Yes	All R5
UK7	IVDU	90	AZT	Severe	Brain	UK7-BR	R5	6	Yes	All R5

The clinical and neuropathological details of the study subjects, and the derivation and characterization of the primary tissue derived HIV-1 isolates have been published previously [14,24,27], and are summarized again here to assist in the interpretation of the data derived from the cloned Envs. Envs were amplified from primary virus isolates by PCR and cloned into the pSVIII-Env expression vector as described previously [29,30,33,35]. Functional Envs were identified by pseudotyping onto Env-deficient GFP reporter virus and entry assays in JC53 cells, as described previously [14,29,30,34,35]. Coreceptor usage of cloned Envs was determined by pseudotyping onto Env-deficient luciferase reporter virus that were generated in 293T cells, and entry assays in Cf2th-CD4 cells expressing CCR5 or CXCR4, as described previously [30,35]. The coreceptor usage of Envs derived from brain and spleen of subject MACS1 has been reported recently [30]. The additional Envs described here have been assigned Genbank accession numbers JN001990 to JN002061. Six functional Envs were cloned from Macs2-BR and UK1-BR viruses, but sequencing and phylogenetic analysis revealed that only 5 and 4 clones, respectively, were independent with unique nucleotide sequences. Thus, only independent Envs are listed here and included for the subsequent structural and functional analyses. MH, male homosexual; IVDU, intravenous drug user; mo, month; ddI, didanosine; AZT, zidovudine; ddC, zalcitabine; CSF, cerebrospinal fluid; PBMC, peripheral blood mononuclear cells; S. Cord, spinal cord; L. Node, lymph node.

### Production of three-dimensional gp120 models and characterization of the CD4bs cavity

We next produced three-dimensional structural models of each of the 81 Envs using a protocol that we described recently [13,32]. Briefly, homology models of CD4-bound gp120 sequences were prepared using the Build Model protocol of the Discovery Studio suite, version 1.6 (Accelrys, San Diego, CA, USA). This approach used the Modeller algorithm to generate an atomic model of the target protein from a template molecule and a sequence alignment. The template-based models were optimized by iterative cycles of conjugate-gradient minimisation against a probability density function that included spatial restraints derived from the template and residue specific properties [37]. The crystal structure of JRFL gp120 containing the V3 variable loop and bound to CD4 and the X5 Fab antibody fragment was used as the template for CD4-bound models [9] (Protein Data Bank ID: 2B4C). The X5 antibody fragment was deleted from the CD4-bound template prior to modeling. The coordinates for gp120 and CD4 were extracted from the 2B4C crystal structure. Sequence alignments were generated between JRFL gp120 and the primary gp120 Env clones. The sequence for CD4 was included as a second polypeptide chain such that the models of gp120 were constructed as complexes with CD4. The V1V2 variable loops were replaced with a GAG linker sequence and the N- and C- termini overhangs were cut using the modeling software.

Similarities in three-dimensional structure were measured by the root mean square deviation (RMSD) of the distances between main-chain atoms (N, Cα, C and O atoms) from crystal and model structures after rigid body superposition, where an RMSD of < 1Å signifies a high level of three-dimensional structural similarity between overlayed proteins. The overall quality of the geometry of gp120 models generated was verified using PROCHECK [38].

**Figure 2:**
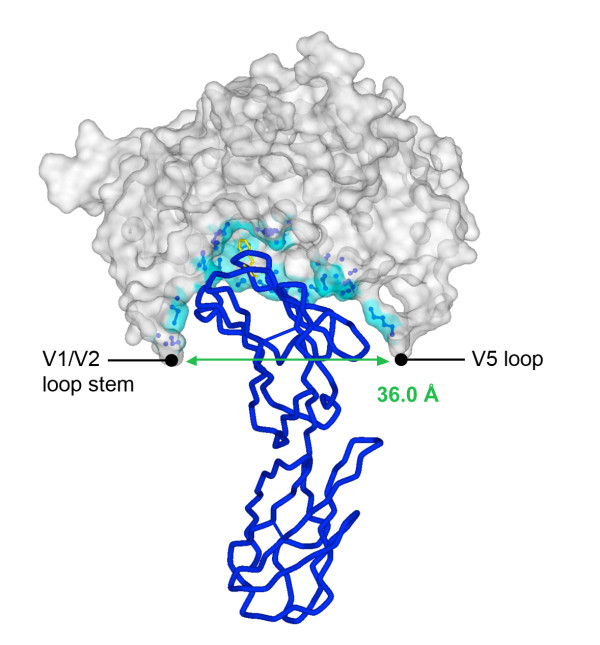
**Predicted alterations in the CD4bs cavity from three-dimensional gp120 models**. The gp120 model of Macs2ln5 Env is shown in molecular surface representation, and the CD4 molecule is shown in blue Cα wire, with Phe43 of CD4 highlighted in yellow stick representation to show the "Phe43 cavity" of gp120. gp120 residues in the CD4 binding pocket located within 4 Å of the CD4 molecule are shown in ball and stick representation and their molecular surface is highlighted in blue. The width of the CD4bs aperture, as constrained by the inner-most atoms at the gp120 V1V2 stem and the V5 loop, was deduced as described previously [13].

The three-dimensional structural similarity between the 2B4C JRFL crystal structure and the 81 predicted structures of the primary gp120 proteins was < 1.0 Å for all the primary gp120 models (data not shown), indicating a high overall degree of structural similarity. Identical RMSD values for each gp120 model were obtained upon repeated, independent modeling operations (data not shown). The aperture width of the CD4bs cavity was deduced from each of the three-dimensional gp120 structural models by measuring the distance between the inner most atoms present at the stem of the V1V2 loops and the V5 loop, which constrain the CD4 binding pocket of gp120 [9]. Figure 2 shows the derivation of the CD4bs aperture width for the Macs2ln5 gp120 model as an example. Analysis of structural models generated for all the 81 Envs showed that the aperture width of the predicted CD4bs cavity ranges from 30 to 36 Å in this panel of Envs.

### An increased aperture width of the gp120 CD4bs cavity is associated with increased fusogenicity, increased CD4-usage, and increased IgG1b12 Env mAb binding

**Figure 3:**
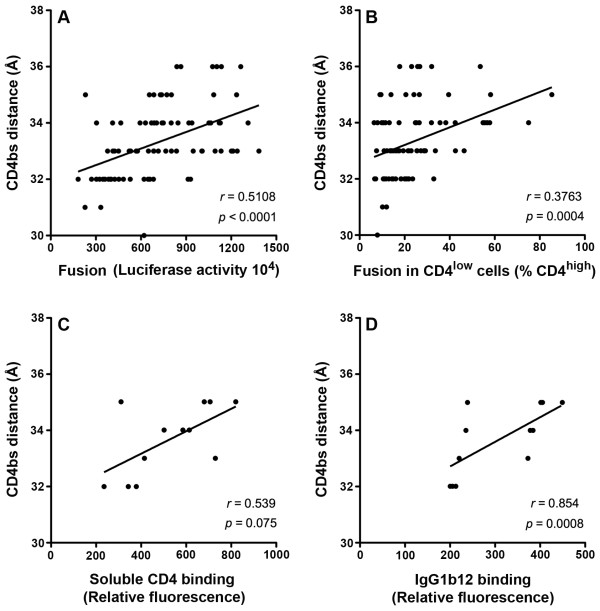
**The effect of gp120 CD4bs cavity alterations on fusogenicity, CD4-dependence, sCD4 binding and CD4bs exposure**. The CD4bs aperture widths for each gp120 model were plotted against the ability of Env to mediate cell-cell fusion (A), the ability of Env to utilize low levels of CD4 for cell-cell fusion (B), and the ability of Env to bind sCD4 (C) or the Env mAb IgG1b12 (D), using Prism version 5.0c (GraphPad Software). The methods for these functional and biochemical assays have been described in detail previously, including the extensive use of controls to ensure equivalent expression of Env on the cell surface, protocols for generating (and measuring CD4 expression on) CD4^low ^and CD4^high ^cells, and the empirical determination of sCD4 and IgG1b12 concentrations used that we showed were within the linear range of Env binding [13,32,35]. The Spearman correlation coefficient (r) and *P *values are shown. *P *values < 0.05 were considered statistically significant. The data shown are representative of 3 independent experiments.

To determine whether alterations in the width of the gp120 CD4bs aperture may influence Env function, we first performed quantitative cell-cell fusion assays with 293T effector cells expressing equivalent levels of Env on the cell surface, and target cells expressing coreceptor and either relatively high or relatively low levels of CD4 as described previously [13]. In these assays, we observed positive correlations between the width of the gp120 CD4bs cavity and the overall level of cell-cell fusion (Figure 3A), and also with the ability of Env to utilize low levels of CD4 to mediate cell-cell fusion (Figure 3B). Next, to better understand the influence of changes in the gp120 CD4bs aperture on CD4 binding and CD4bs exposure, we measured the ability of Env to bind sCD4 and the Env mAb IgG1b12, whose epitope overlaps the gp120 CD4bs, using a subset of Envs (n = 12, Macs1br2-8 and Macs1sp3-15; see Figure 1), as described previously [13,35]. In these assays, we observed a near significant association between the width of the CD4bs cavity and ability of Env expressed on 293T cells to bind sCD4 (Figure 3C), and a significant correlation between this parameter and the ability of Env to bind IgG1b12 (Figure 3D). Together, these studies, using a large and newly-described panel of primary Envs, demonstrate the utility of three-dimensional modeling of the gp120 CD4bs cavity for better understanding the structural basis of Env-CD4 interactions, confirming the results of our recent study of a different and much smaller panel of Envs [13]. Furthermore, our results provide new evidence suggesting that these predicted CD4bs conformational alterations augment the ability of gp120 to interact with CD4 by increasing the exposure of the CD4bs.

### Repositioning of the V5 loop is associated with conformational alterations in the gp120 CD4bs

**Figure 4:**
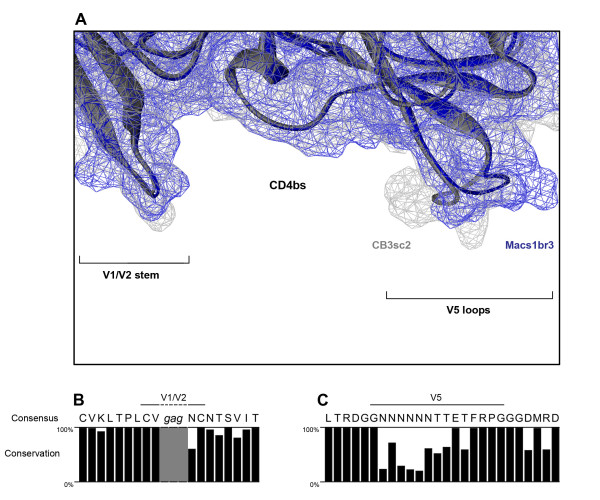
**Repositioning of the V5 loop is associated with structural alterations in the CD4bs cavity**. The gp120 models of CB3sc2 and Macs1br3 Envs (grey and blue ribbon representation, respectively) were superimposed, and their molecular surfaces were presented as blue or grey wire mesh (A). The consensus V1V2 stem (B) and V5 loop sequence (C) was deduced for all 81 primary Envs, and the degree of conservation at each amino acid position was calculated. The GAG linker sequence, which replaced the V1V2 loops in the crystal and model structures, is shown and highlighted as a grey box in panel (B).

To elucidate the gp120 determinants which may contribute to structural alterations in the CD4bs and which subsequently influence CD4 interactions and fusogenicity, we next compared the structural similarity between CB3sc2 and Macs1br3 gp120 models which are predicted to have the narrowest and widest of the CD4bs apertures (30 and 36 Å, respectively). Overlays of these molecular models revealed a high degree of structural similarity within the V1V2 stem region, but notable structural variation within the V5 loop region (Figure 4A). Furthermore, sequence analysis of all the primary Env clones showed a relatively high degree of sequence homology within the V1V2 stem region (Figure 4B), but a relatively high degree of sequence variation within the V5 loop (Figure 4C). Together, these results suggest that, in this new panel of Envs, alteration in the width of the CD4bs cavity is likely to be due to sequence variability within the V5 region of gp120 which repositions the V5 loop.

## Conclusions

Using a large, independent panel of Envs, we confirmed that structural alterations in the gp120 CD4bs can be deduced using optimized three-dimensional gp120 molecular models, and that these alterations may influence fusogenicity and the ability of gp120 to interact with CD4. We further show, for the first time, that these alterations appear to increase the exposure of the CD4bs. Thus, our study provides new insights into structural mechanisms that contribute to altered interactions between gp120 and CD4. These insights contribute to a better understanding of HIV-1 entry and in addition, may inform the design of Env vaccine immunogens where enhanced exposure of the CD4bs may be desirable to elicit effective neutralizing antibody responses. Furthermore, our modeling approach may be informative for better understanding structural mechanisms contributing to HIV-1 disease progression. Here, we finally describe and characterize a new and relatively large panel of functional Envs from brain and other tissues, which will enhance the capacity of investigators to undertake NeuroAIDS research.

## List of abbreviations used

HIV-1: Human immunodeficiency virus type 1; SIV: Simian immunodeficiency virus; SHIV: Simian-human immunodeficiency virus; Env: HIV-1 envelope glycoproteins; CD4bs: CD4 binding site; GFP: Green fluorescent protein; RMSD: Room mean squared deviation; sCD4: soluble CD4; mAb: monoclonal antibody; BR: Brain; CSF: Cerebrospinal fluid; PBMC: Peripheral blood mononuclear cells; SC: Spinal cord; Spln: Spleen; LN: Lymph node

## Competing interests

The authors declare that they have no competing interests.

## Authors' contributions

LG, JS and PRG designed the experiments. LG and JS performed the experiments. JS and PAR designed the molecular models and interpreted the modeling data. MJC assisted with Env cloning and sequencing, and helped interpret the results. PRG supervised the project and helped interpret the results. LG and PRG wrote the manuscript. All authors helped edit the manuscript and have read and approved the final version.
